# A Dictionary of Terms Employed by the French, in Anatomy, Physiology, Pathology, Practical Medicine, Surgery, Midwifery, Pharmacy, Medical Zoology, Botany, and Chemistry, &c.

**Published:** 1834-10-01

**Authors:** 


*A Dictionary of Terms employed by the French, in Anatomy, Physiology, Pathology, Practical Medicine, Surgery, Midwifery, Pharmacy, Medical Zoology, Botany, and Chemistry; with their Derivations from the Greek, Latin, French, German, and English; Explanations in English; and Illustrations in different Languages.* By SHIRLEY PALMER, M.D., formerly of Tamworth. Part I.—Birmingham, 1834. 8vo. pp. 160.

WHEN you go into company, gentle reader, you will often hear people talking of the possibility of learning French, or even German, in a few months: nay, you will sometimes meet a Thraso who has the face to say that he has done so himself. Should such things be uttered in your presence, we recommend you, if you be a man of tact, and desirous to get on in the world, not to cry out, as Dr. Johnson used to do, "No, sir; it is not so; what you say is otherwise:" rest satisfied with thinking this, and, like the heroine of a novel, utter silent ejaculations. If this millennium of languages is ever to arrive, when French and German may be acquired by a lawyer in the long vacation, we are certain that it must be preceded by a very prodigious improvement in the present dictionaries; and we hail this technical lexicon, by Dr. Palmer, as a first step towards the construction of the great verbal railway. Had any one, before the appearance of this work, inquired where he might find an explanation of French scientific terms, we should have referred him to the *Vocabulaire de Wailly*, or the *Supplément au Dictionnaire de l'Académie*, (Paris, 1827, 4to. pp. 559.) When our author's dictionary, however, is completed, it will be far preferable; partly because Dr. Palmer, having confined himself to a few sciences, has room for more copious explanations, and partly because the other works, being in French, are useful to those only who have made considerable progress in the language. Moreover, it will be an attraction to many students, that our author gives the German synonyms; and, as he promises an index of them, on the completion of the work, it will become in fact a German dictionary also. This will be an especial lift to our medical brethren; for, though they have been nibbling at German pretty extensively for the last ten years, we fear there have been but few bites: indeed, if we were to make a guess at the number of doctors who can read German with fluency, our opinion might be thought impolite, and unworthy of a place in this, the most urbane of Journals; and we will therefore not even hint our suspicions.

Two or three quotations will show our readers the plan of the work.

"A and āā represent, in medical prescription, the Greek ἀνὰ, (French *de chaque*, and German *von jedem*,) of each. In Latin, the adject. singulorum or —arum, according to the gender of the preceding nouns, is more precise and elegant. *Exs.* Extracti Anthemidis,—Pulveris Scillæ, *singulorum* ʒ1. Tincturæ Sennæ,—Tinct. Aloës, *singularum* ʒ1. In French formulæ, the *āā*,—in German, the *ana*,—is commonly employed.

"Abaissement, s. m. —depressio, defectio, f. L.—herabziehung, niederziehung, f., niedersinken, n., niederschlagung, f. G.,—lowering; sinking or falling down; depression; failure, lowness. *Exs.* 1. An effect of the action of depressor muscles: *Abaissement* du bras, *lowering* of the arm.—2. The condition of a part or organ sinking from its own weight: — de l'uterus, *falling down* (prolapsus) of the womb.—3. A mode of surgical operation for cataract: operation de la cataracte par *abaissement*, operation for cataract by *depression*.—4. A mental affection resulting from the influence of physical or moral causes: — de courage, — defectio animi, L.,—niedergeschlagenheit, f. G.,—*depression*—*failure*—*lowness*—of spirits.

"Abaisseur, s. m., and adj.,—depressor: a name given to those muscles (musculi depressores, L.---niederziehende muskeln, G.), whose function consists in *depressing* the organ or part to which their moveable extremity is attached. *Exs.*

"1. — *de l'œil*, depressor of the eye. See Droit *inférieur* —.

"2. --- *de la mâchoire inférieure*, depressor of the lower jaw. See Digastrique.

"3. — *de l'aile du nez*, (alveolo-nasal, Chaussier),---depressor, alæ nasi, myrtiformis, L.,---herabzieher des nasenflügels, G.,---depressor of the ala nasi: a pair of muscles extending from the vicinity of the anterior nasal spine to the posterior region of the corresponding ala nasi.

"4. ---*de l'angle des lèvres*, (maxillo-labial, Ch.)---depressor anguli oris, triangularis menti, L.,---herabzieher des mundwinkels, G.,---depressor of the angle of the mouth: a triangular muscle extending, on each side, from the external oblique line of the lower jaw to the commissure of the lips.

"5. --- (*carré*) *de la lèvre inférieure*, carré du menton, (mento-labial, Ch.)---depressor labii inferioris, quadratus menti, L.---herabzieher der unterlippe, G.,---depressor of the lower lip: a square muscle extending, on each side, from the external maxillary line to the lower lip.

"Abaisseur *de la langue*,—γλωσσοκάτοχος,—linguæ depressor: an instrument wherewith to *depress* the *tongue*, instead of the spoon ordinarily employed, in examination of the fauces." (P. 1.)

There seems to us to be a relationship between the *ana* of prescriptions and one sense of the Greek ἀνὰ, but not quite so clear a one as the writers of medical lexicons would have us believe. Thus, when Xenophon talks of a journey being performed ἀνα πεντε παρασαγγας της ἡμερας, at the rate of five parasangs a day, we may imagine a kind of equal distribution of parasangs over the whole time occupied in the journey, and hence that *ανα* came to be taken by the prescribers to signify equality. The mark (there should be but one,) over the āā is a substitute for the n, and has done duty for that letter for many centuries.*

"Céphalée, s. f.,—κεφαλαία,—cephalæa, f. G.: correctly a chronic—employed by some writers as expressive of very violent and obstinate—*headach:* frequently, as a synonym of Céphalalgie.

"Céphaline, s. f.: a term borrowed from the *Greek*, κεφαλίνη; and signifying the base or root of the tongue.

"Céphalique, adj.,—κεφαλικὸς,—cephalicus, L.,—cephalic, belonging to the *head:* an epithet, in anatomy, applied by Chaussier to the primitive *carotid artery*,—*Artère cephalique*, F.,—kopfschlagader, G.; and to the internal *jugular vein*,—*veine céphalique*. The latter term has commonly been assigned to a

* In Greek mss. the line stands for *ν*, if written over a vowel, and for *α*, if over a consonant. (*Porson in Hec.* v. 1161.) In Portuguese, the n often has a waved line over it, (the *tilde*,) which alters the pronunciation, and may therefore be considered as constituting another letter. Want of attention to this made Lord Byron take *pena* with the line for *pena* without the line, the former signifying *rock*, and the latter *punishment*. See *Childe Harold*, Canto i. stanza xx., and his two notes upon it.

cutaneous vein (radiale cutanée, Ch.), situated on the outer side of the thoracic limb; from which the ancients were accustomed to take blood in *cephalic* affections: probably influenced, in this practice, by observing that the vessel almost invariably anastomozed with the external jugular vein. In materia medica, the term *Céphaliques*, pl., is applied to remedies—κεφαλικὰ φάρμακα,—remedia *cephalica*, L., *haupt*mittel, G.,—prescribed for the removal of affections of the *head*." (P. 120.)

" Corde, s. f., (χορδὴ, the string of a bow, or musical instrument, made of gut),—chorda, f., funiculus, m. L.,—strick, m., saite, f. G.,—chord or cord: a term in anatomy, applied, 1., by the ancients, to *tendons* in general; and, especially, to the great tendon of the leg, *corde d'Hippocrate*, F.,—tendo Achillis, L.,—see Tendon: 2. to a slender *nerve* of the internal *ear*,—la *corde du tympan*, ou *du tambour* (filet tympanique du nerf facial, Ch.), F., — *chorda* —*funiculus*---tympani, L., --- paukenfell*saite*, G.;--- which, quitting the facial nerve in the aquæductus Fallopii, enters the cavity of the tympanum, by an orifice situated above the pyramid; and, passing between the longer process of the incus and the handle of the malleus, goes out through the fissura Glasseri, to unite, at an acute angle, with the lingual branch of the inferior maxillary nerve, and thus establish a communication between the spheno-palatine and submaxillary ganglia; and 3. to the *ligaments* of the *rima glottidis*,---*cordes vocales*, F.,---*chordæ* vocales, L.,— stimmritzen*bänder*, G.,---which some anatomists have regarded as tense *chords* producing the phenomena of voice.

" In certain cases of acute blennorrhagia, or urethritis, the spongy structure of the male urethra forms an indurated and knotty *tumour*,—in *French* pathology termed *corde*,—of oblong figure, beneath the membrum virile, and opposing the elongation of that organ in the state of erethism. This morbid condition constitutes the affection named *Cordée chaudepisse* by the *French*,—the harnstrenge of *German* writers." (P. 159.)

The part before us extends from A to Corne: the second part is to appear about December, and the third next spring. When this work is completed, it will not only find a place in every well-selected medical library, but it will be a most valuable aid to the young student, and save him many a weary hunt for a hard word.

